# Crystal structure of 4-amino-5-chloro-2,6-di­methyl­pyrimidinium thio­phene-2,5-di­carboxyl­ate

**DOI:** 10.1107/S2056989016010148

**Published:** 2016-06-24

**Authors:** Ammaiyappan Rajam, Packianathan Thomas Muthiah, Ray J. Butcher, Matthias Zeller

**Affiliations:** a School of Chemistry, Bharathidasan University, Tiruchirappalli 620 024, Tamilnadu, India; b Department of Chemistry, Howard University, 525 College Street NW, Washington, DC 20059, USA; c Department of Chemistry, Youngstown State University, 1 University Plaza, Youngstown, OH 44555, USA

**Keywords:** crystal structure, crystal salts, hydrogen-bonding patterns, base-pairing, π–π stacking, C—H⋯π inter­actions

## Abstract

In the title salt, C_6_H_9_ClN_3_^+^·C_6_H_3_O_4_S^−^, the cations and anions are linked *via* O—H⋯O and N—H⋯O hydrogen bonds, forming *R*_6_^6^(37) ring motifs that are inter­connected with each other, producing sheets. Separate parallel inversion-related sheets are linked through N—H⋯N and π–π stacking inter­actions [centroid–centroid distance = 3.5414 (13) Å], forming double layers parallel to (101). Weak C—H⋯O and C—H⋯S hydrogen bonds, as well as C—H⋯π inter­actions, connect the double layers into a three-dimensional network.

## Abstract 2

In the title salt, the cations and anions are linked through O—H⋯O,N—H⋯O, N—H⋯N and π–π stacking inter­actions, forming double layers parallel to (101). Weak C—H⋯O and C—H⋯S hydrogen bonds connect the double layers into a three-dimensional network.

## Chemical context   

In crystal engineering, non-covalent inter­actions, such as hydrogen bonding, play a key role in mol­ecular recognition processes (Desiraju, 1989 ▸). Pyrimidine derivatives have gained considerable importance because of their remarkable bio­logical properties, for example as anti-fungal, anti­viral, anti­cancer and anti-allergenic agents (Ding *et al.*, 2004 ▸). Thio­phene­carb­oxy­lic acid and its derivatives have attracted attention because of their wide range of pharmacological properties and numerous applications, such as the preparation of DNA hybridization indicators, single-mol­ecule magnets, photoluminescence materials and the treatment of osteoporosis as inhibitors of bone resorption in the tissue culture (Bharti *et al.*, 2003 ▸; Taş *et al.*, 2014 ▸; Boulsourani *et al.*, 2011 ▸). The present study investigates the hydrogen-bonding patterns in 4-amino-5-chloro-2,6-di­methyl­pyrimidinium thio­phene-2,5-di­carboxyl­ate (I).
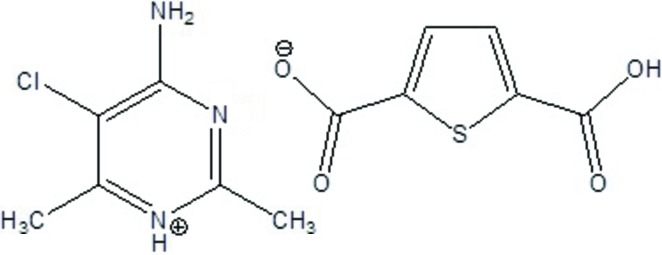


## Structural commentary   

**Figure 1:**
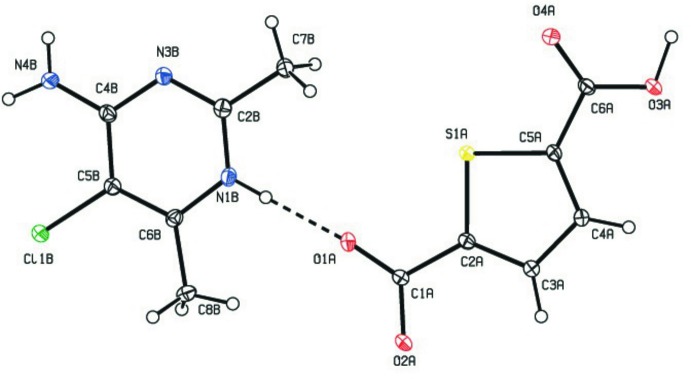
The asymmetric unit of the title compound, showing 30% probability displacement ellipsoids. The dashed line indicates a hydrogen bond.

The asymmetric unit of C_6_H_9_ClN_3_^+^·C_6_H_3_O_4_S^−^, (I), contains one 4-amino-5-chloro-2,6-di­methyl­pyrimidinium cation and one thio­phene-2,5-di­carboxyl­ate anion (Fig. 1 ▸). Protonation of the pyrimidine occurs at atom N1, leading to a C2*B*—N1*B*—C6*B* angle of 122.5 (2)° which an increase of *ca* 3.8° compared to the C2*B*—N3*B*—C4*B* angle 118.7 (2)° involving the unprotonated N3 atom.

## Supra­molecular features   

**Figure 2:**
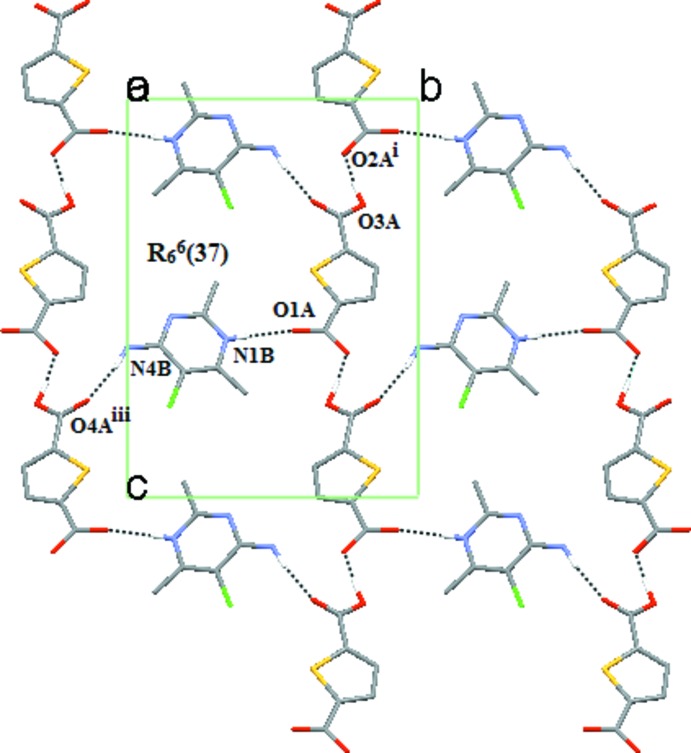
Packing diagram for (I), viewed along the *a* axis, showing a single sheet formed by O—H⋯O and N—H⋯O hydrogen bonds. Symmetry codes are given in Table 1 ▸. Dashed lines represent hydrogen bonds.

**Figure 3:**
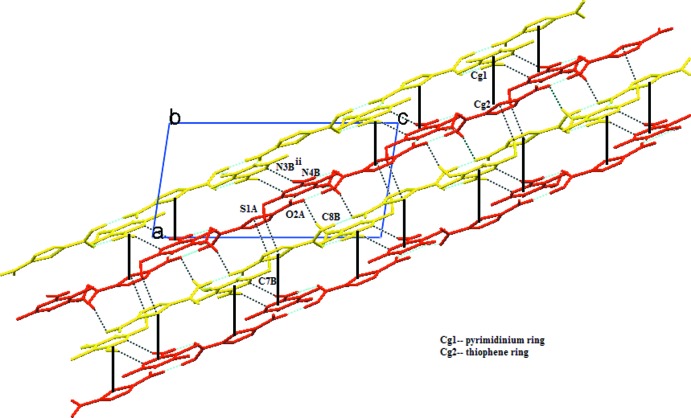
A view along the *b* axis, showing double layers (indicated in red and yellow) formed by hydrogen bonds and π–π stacking inter­actions. The weak C—H⋯O and C—H⋯S hydrogen bonds connect the double layers to form a three-dimensional network. Dotted lines represent N—H⋯N, C—H⋯O and C—H⋯S inter­actions. Solid lines indicate the stacking inter­actions.

The carboxyl­ate group of the thio­phene-2,5-di­carboxyl­ate anion inter­acts with the protonated N1 atom of the pyrimidinium moiety with a single point heterosynthon *via* N—H⋯O hydrogen bonds (Table 1 ▸). In addition, the components are connected through O—H⋯O and N—H⋯O hydrogen bonds (Table 1 ▸) to form an 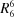
(37) ring graph set motif. This motif includes anions connected by O—H.·O hydrogen bonds along [10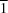] and involves the cations along [010] to form a 2D sheet (Fig. 2 ▸). Two separate 2D sheets (which are indicated in red and yellow in Fig. 3 ▸) are inter­connected by a self-complementary base pair between the pyrimidinium moiety through N—H⋯N hydrogen bond inter­actions with an 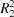
(8) ring graph set motif and π–π stacking inter­actions between the pyrimidinium ring and the thio­phene ring with an observed inter­planar distance of 3.4188 (10) Å, a centroid-to-centroid (*Cg*1–*Cg*2) distance of 3.5414 (13) Å (where *Cg*1 is the centroid of the ring N1*B*/C2*B*–C6*B* and *Cg*2 is the centroid of the ring S1*A*/C2*A*–C5*A*) and slip angle (the angle between the centroid vector and the normal to the plane) of 18.0°; these are typical aromatic stacking values (Hunter, 1994 ▸). Through these inter­actions, parallel inversion-related sheets are connected into double layers parallel to (101). In addition, weak C—H⋯O, C—H⋯S and C—H⋯π inter­molecular inter­actions connect the double layers into a three-dimensional network (Fig. 3 ▸).

## Database survey   

The crystal structures of amino­pyrimidine derivatives (Schwalbe & Williams, 1982 ▸) and amino­pyrimidine carboxyl­ates (Hu *et al.*, 2002 ▸), have been reported. Several co-crystals/salts of amino­pyrimidine derivatives have been reported from our laboratory including co-crystals/salts of amino­pyrimidines with carb­oxy­lic acid (Mu­thiah *et al.*, 2006 ▸; Devi & Mu­thiah, 2007 ▸; Subashini *et al.*, 2008 ▸; Thanigaimani *et al.*, 2009 ▸; Ebenezer & Mu­thiah, 2010 ▸, 2012 ▸; Ebenezer *et al.*, 2011 ▸), amino­pyrimidines–thio­phene­carb­oxy­lic acid (Jegan Jennifer *et al.*, 2014 ▸), the crystal structure of 2-amino-4,6-di­meth­oxy­pyrimidiniumthio­phene-2-carboxyl­ate (Rajam *et al.*, 2015 ▸) and metal complexes with 4-amino-5-chloro-2,6-di­methyl­pyrimidine (Karthikeyan *et al.*, 2016 ▸)

## Synthesis and crystallization   

A hot DMF solution of 4-amino-5-chloro-2,6-di­methyl­pyrimidine (39 mg, Alfa Aesar) and thio­phene-2,5-di­carb­oxy­lic acid (43 mg, Alfa Aesar) were mixed and warmed for half an hour over a water bath. The mixture was cooled slowly and kept at room temperature. After a few days colourless plate-like crystals were obtained.

## Refinement   

Crystal data, data collection and structure refinement details are summarized in Table 2 ▸. The N—H and O—H H atoms were located in difference Fourier maps and refined isotropically. All other H atoms were placed in calculated positions and refined using a riding-model approximation with C—H = 0.95 Å (CH) or 0.98 Å (CH_3_). Isotropic displacement parameters for these atoms were set to 1.2 (CH) or 1.5 (CH_3_) times *U*_eq_ of the parent atom. Idealized Me H atoms were refined as rotating groups. There are larger than expected residual density peaks close to the Cl and S atoms but these are not chemically sensible and are assumed to be related to the quality of the crystal.

## Supplementary Material

Crystal structure: contains datablock(s) I. DOI: 10.1107/S2056989016010148/lh5814sup1.cif

Structure factors: contains datablock(s) I. DOI: 10.1107/S2056989016010148/lh5814Isup2.hkl

Supporting information file. DOI: 10.1107/S2056989016010148/lh5814Isup3.cml

CCDC reference: 1486940

Additional supporting information: crystallographic information; 3D view; checkCIF report

## Figures and Tables

**Table 1:** Hydrogen-bond geometry (Å, °) *Cg* is the centroid of the S1*A*/C2*A*–C5*A* ring.

*D*—H⋯*A*	*D*—H	H⋯*A*	*D*⋯*A*	*D*—H⋯*A*
O3*A*—H3*A*⋯O2*A* ^i^	1.04 (4)	1.44 (4)	2.475 (2)	176 (4)
N1*B*—H1*B*⋯O1*A*	0.85 (3)	1.87 (3)	2.719 (3)	178 (3)
N4*B*—H4*B*1⋯N3*B* ^ii^	0.86 (3)	2.40 (3)	3.218 (3)	158 (3)
N4*B*—H4*B*2⋯O4*A* ^iii^	0.94 (3)	1.86 (3)	2.784 (3)	170 (3)
C7*B*—H7*BB*⋯S1*A* ^iv^	0.98	2.86	3.807 (2)	164
C8*B*—H8*BB*⋯O3*A* ^v^	0.98	2.53	3.281 (3)	134
C8*B*—H8*BC*⋯O2*A* ^vi^	0.98	2.47	3.301 (3)	143
C7*B*—H7*BB*⋯*Cg* ^iv^	0.98	2.69	3.556 (3)	148

Symmetry codes: (i) 
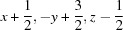
; (ii) 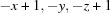; (iii) 
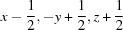
; (iv) 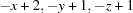; (v) 
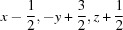
; (vi) 
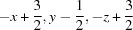
.

**Table 2:** Experimental details

Crystal data	
Chemical formula	C_6_H_9_ClN_3_^+^·C_6_H_3_O_4_S^−^
*M*_r_	329.76
Crystal system, space group	Monoclinic, *P*2_1_/*n*
Temperature (K)	100
*a*, *b*, *c* (Å)	7.9948 (3), 11.3928 (4), 15.7757 (6)
β (°)	98.520 (2)
*V* (Å^3^)	1421.04 (9)
*Z*	4
Radiation type	Mo *K*α
μ (mm^−1^)	0.44
Crystal size (mm)	0.23 × 0.19 × 0.06

Data collection	
Diffractometer	Bruker AXS D8 Quest CMOS
Absorption correction	Multi-scan (*SADABS*; Krause *et al.*, 2015 ▸)
*T*_min_, *T*_max_	0.424, 0.746
No. of measured, independent and observed [*I* > 2σ(*I*)] reflections	10749, 3911, 2862
*R*_int_	0.053
(sin θ/λ)_max_ (Å^−1^)	0.704

Refinement	
*R*[*F* ^2^ > 2σ(*F* ^2^)], *wR*(*F* ^2^), *S*	0.060, 0.185, 1.10
No. of reflections	3911
No. of parameters	208
H-atom treatment	H atoms treated by a mixture of independent and constrained refinement
Δρ_max_, Δρ_min_ (e Å^−3^)	1.59, −0.69

Computer programs: *APEX2* and *SAINT* (Bruker, 2014 ▸), *SIR92* (Altomare *et al.*, 1993 ▸), *SHELXL2014* (Sheldrick, 2015 ▸), *SHELXLE* (Hübschle *et al.*, 2011 ▸), *Mercury* (Macrae *et al.*, 2008 ▸) and *PLATON* (Spek, 2009 ▸).

## supplementary crystallographic information

### Crystal data

**Table:**

C_6_H_9_ClN_3_^+^·C_6_H_3_O_4_S^−^	*F*(000) = 680
*M**_r_* = 329.76	*D*_x_ = 1.541 Mg m^−^^3^
Monoclinic, *P*2_1_/*n*	Mo *K*α radiation, λ = 0.71073 Å
*a* = 7.9948 (3) Å	Cell parameters from 6601 reflections
*b* = 11.3928 (4) Å	θ = 3.1–30.0°
*c* = 15.7757 (6) Å	µ = 0.44 mm^−^^1^
β = 98.520 (2)°	*T* = 100 K
*V* = 1421.04 (9) Å^3^	Plate, colourless
*Z* = 4	0.23 × 0.19 × 0.06 mm

### Data collection

**Table:**

Bruker AXS D8 Quest CMOS diffractometer	3911 independent reflections
Radiation source: I-mu-S microsource X-ray tube	2862 reflections with *I* > 2σ(*I*)
Laterally graded multilayer (Goebel) mirror monochromator	*R*_int_ = 0.053
ω and φ scans	θ_max_ = 30.0°, θ_min_ = 2.6°
Absorption correction: multi-scan (SADABS; Krause *et al.*, 2015)	*h* = −11→9
*T*_min_ = 0.424, *T*_max_ = 0.746	*k* = −15→16
10749 measured reflections	*l* = −21→21

### Refinement

**Table:**

Refinement on *F*^2^	Primary atom site location: structure-invariant direct methods
Least-squares matrix: full	Secondary atom site location: difference Fourier map
*R*[*F*^2^ > 2σ(*F*^2^)] = 0.060	Hydrogen site location: mixed
*wR*(*F*^2^) = 0.185	H atoms treated by a mixture of independent and constrained refinement
*S* = 1.10	*w* = 1/[σ^2^(*F*_o_^2^) + (0.1146*P*)^2^] where *P* = (*F*_o_^2^ + 2*F*_c_^2^)/3
3911 reflections	(Δ/σ)_max_ < 0.001
208 parameters	Δρ_max_ = 1.59 e Å^−^^3^
0 restraints	Δρ_min_ = −0.69 e Å^−^^3^

### Special details

**Table:**

Geometry. All esds (except the esd in the dihedral angle between two l.s. planes) are estimated using the full covariance matrix. The cell esds are taken into account individually in the estimation of esds in distances, angles and torsion angles; correlations between esds in cell parameters are only used when they are defined by crystal symmetry. An approximate (isotropic) treatment of cell esds is used for estimating esds involving l.s. planes.

### Fractional atomic coordinates and isotropic or equivalent isotropic displacement parameters (Å^2^)

**Table:**

	*x*	*y*	*z*	*U*_iso_*/*U*_eq_	
S1A	0.83375 (7)	0.63795 (4)	0.43028 (4)	0.01808 (18)	
O1A	0.6790 (2)	0.57301 (14)	0.58125 (10)	0.0252 (4)	
O2A	0.6743 (2)	0.75418 (14)	0.63699 (10)	0.0218 (4)	
O3A	1.0617 (2)	0.81066 (15)	0.26723 (10)	0.0227 (4)	
H3A	1.110 (5)	0.780 (3)	0.214 (2)	0.060 (11)*	
O4A	0.9244 (3)	0.63757 (15)	0.25678 (12)	0.0309 (4)	
C1A	0.7104 (3)	0.6802 (2)	0.58237 (14)	0.0178 (4)	
C2A	0.7953 (3)	0.7287 (2)	0.51203 (14)	0.0187 (4)	
C3A	0.8446 (3)	0.84283 (19)	0.49963 (15)	0.0204 (5)	
H3AA	0.8325	0.9054	0.5381	0.024*	
C4A	0.9151 (3)	0.85594 (18)	0.42313 (15)	0.0199 (5)	
H4AA	0.9568	0.9282	0.4046	0.024*	
C5A	0.9166 (3)	0.75252 (19)	0.37866 (14)	0.0182 (4)	
C6A	0.9689 (3)	0.7288 (2)	0.29484 (14)	0.0198 (5)	
Cl1B	0.43554 (7)	0.14586 (5)	0.77201 (3)	0.02236 (18)	
N1B	0.6246 (2)	0.34003 (17)	0.60286 (13)	0.0206 (4)	
H1B	0.639 (4)	0.413 (3)	0.5956 (19)	0.031 (7)*	
N3B	0.6059 (2)	0.14982 (16)	0.54703 (13)	0.0205 (4)	
N4B	0.5127 (3)	−0.00013 (17)	0.62434 (14)	0.0242 (4)	
H4B1	0.512 (4)	−0.043 (3)	0.5793 (19)	0.040 (9)*	
H4B2	0.478 (4)	−0.039 (3)	0.671 (2)	0.045 (9)*	
C2B	0.6450 (3)	0.2616 (2)	0.54118 (15)	0.0204 (5)	
C4B	0.5460 (3)	0.1121 (2)	0.61845 (15)	0.0205 (5)	
C5B	0.5201 (3)	0.1938 (2)	0.68398 (14)	0.0196 (4)	
C6B	0.5592 (3)	0.3099 (2)	0.67489 (15)	0.0191 (4)	
C7B	0.7142 (3)	0.3044 (2)	0.46395 (15)	0.0235 (5)	
H7BA	0.6941	0.2453	0.4185	0.035*	
H7BB	0.8360	0.3181	0.4787	0.035*	
H7BC	0.6579	0.3778	0.4439	0.035*	
C8B	0.5357 (3)	0.4046 (2)	0.73702 (15)	0.0250 (5)	
H8BA	0.4198	0.4019	0.7502	0.037*	
H8BB	0.5563	0.4810	0.7120	0.037*	
H8BC	0.6156	0.3931	0.7898	0.037*	

### Atomic displacement parameters (Å^2^)

**Table:**

	*U*^11^	*U*^22^	*U*^33^	*U*^12^	*U*^13^	*U*^23^
S1A	0.0222 (3)	0.0148 (3)	0.0193 (3)	−0.00038 (18)	0.0096 (2)	−0.00055 (19)
O1A	0.0324 (9)	0.0188 (8)	0.0273 (9)	−0.0044 (7)	0.0142 (7)	0.0021 (7)
O2A	0.0242 (8)	0.0230 (8)	0.0207 (8)	−0.0008 (6)	0.0118 (6)	−0.0003 (6)
O3A	0.0267 (8)	0.0229 (8)	0.0217 (8)	−0.0026 (7)	0.0140 (7)	−0.0021 (7)
O4A	0.0445 (11)	0.0221 (9)	0.0308 (10)	−0.0068 (7)	0.0212 (8)	−0.0064 (7)
C1A	0.0186 (9)	0.0192 (10)	0.0164 (10)	0.0001 (8)	0.0054 (8)	−0.0009 (8)
C2A	0.0169 (9)	0.0197 (10)	0.0207 (10)	0.0010 (8)	0.0064 (8)	−0.0006 (9)
C3A	0.0234 (11)	0.0193 (10)	0.0199 (11)	−0.0029 (8)	0.0078 (9)	−0.0015 (8)
C4A	0.0208 (10)	0.0183 (11)	0.0218 (11)	−0.0040 (8)	0.0076 (9)	−0.0013 (8)
C5A	0.0166 (9)	0.0184 (10)	0.0210 (11)	−0.0010 (8)	0.0081 (8)	−0.0002 (8)
C6A	0.0212 (10)	0.0203 (11)	0.0197 (11)	0.0030 (8)	0.0089 (8)	0.0015 (9)
Cl1B	0.0281 (3)	0.0207 (3)	0.0200 (3)	−0.0013 (2)	0.0090 (2)	−0.0002 (2)
N1B	0.0218 (9)	0.0168 (9)	0.0239 (10)	−0.0020 (7)	0.0055 (8)	0.0024 (8)
N3B	0.0225 (9)	0.0188 (10)	0.0212 (10)	0.0004 (7)	0.0059 (8)	0.0018 (7)
N4B	0.0349 (11)	0.0171 (10)	0.0227 (10)	0.0003 (8)	0.0116 (9)	0.0000 (8)
C2B	0.0165 (9)	0.0210 (11)	0.0235 (11)	0.0009 (8)	0.0021 (8)	0.0015 (9)
C4B	0.0183 (10)	0.0215 (11)	0.0228 (11)	0.0013 (8)	0.0071 (8)	0.0006 (9)
C5B	0.0201 (10)	0.0187 (10)	0.0207 (11)	0.0003 (8)	0.0050 (8)	0.0002 (9)
C6B	0.0176 (9)	0.0163 (10)	0.0233 (11)	0.0006 (8)	0.0026 (8)	−0.0003 (9)
C7B	0.0240 (10)	0.0208 (11)	0.0273 (12)	0.0000 (9)	0.0094 (9)	0.0024 (10)
C8B	0.0327 (12)	0.0177 (11)	0.0256 (12)	0.0009 (9)	0.0078 (10)	−0.0044 (9)

### Geometric parameters (Å, º)

**Table:**

S1A—C2A	1.716 (2)	N1B—H1B	0.85 (3)
S1A—C5A	1.722 (2)	N3B—C2B	1.318 (3)
O1A—C1A	1.247 (3)	N3B—C4B	1.358 (3)
O2A—C1A	1.269 (3)	N4B—C4B	1.312 (3)
O3A—C6A	1.306 (3)	N4B—H4B1	0.86 (3)
O3A—H3A	1.04 (4)	N4B—H4B2	0.94 (3)
O4A—C6A	1.226 (3)	C2B—C7B	1.492 (3)
C1A—C2A	1.490 (3)	C4B—C5B	1.429 (3)
C2A—C3A	1.381 (3)	C5B—C6B	1.372 (3)
C3A—C4A	1.414 (3)	C6B—C8B	1.488 (3)
C3A—H3AA	0.9500	C7B—H7BA	0.9800
C4A—C5A	1.372 (3)	C7B—H7BB	0.9800
C4A—H4AA	0.9500	C7B—H7BC	0.9800
C5A—C6A	1.470 (3)	C8B—H8BA	0.9800
Cl1B—C5B	1.721 (2)	C8B—H8BB	0.9800
N1B—C2B	1.348 (3)	C8B—H8BC	0.9800
N1B—C6B	1.363 (3)		
			
C2A—S1A—C5A	91.32 (10)	H4B1—N4B—H4B2	115 (3)
C6A—O3A—H3A	109 (2)	N3B—C2B—N1B	122.3 (2)
O1A—C1A—O2A	126.5 (2)	N3B—C2B—C7B	119.5 (2)
O1A—C1A—C2A	117.76 (19)	N1B—C2B—C7B	118.2 (2)
O2A—C1A—C2A	115.72 (19)	N4B—C4B—N3B	117.9 (2)
C3A—C2A—C1A	128.7 (2)	N4B—C4B—C5B	122.1 (2)
C3A—C2A—S1A	111.97 (17)	N3B—C4B—C5B	120.0 (2)
C1A—C2A—S1A	119.24 (16)	C6B—C5B—C4B	119.5 (2)
C2A—C3A—C4A	112.3 (2)	C6B—C5B—Cl1B	120.89 (18)
C2A—C3A—H3AA	123.9	C4B—C5B—Cl1B	119.55 (17)
C4A—C3A—H3AA	123.9	N1B—C6B—C5B	116.8 (2)
C5A—C4A—C3A	112.36 (19)	N1B—C6B—C8B	117.9 (2)
C5A—C4A—H4AA	123.8	C5B—C6B—C8B	125.2 (2)
C3A—C4A—H4AA	123.8	C2B—C7B—H7BA	109.5
C4A—C5A—C6A	130.0 (2)	C2B—C7B—H7BB	109.5
C4A—C5A—S1A	112.08 (16)	H7BA—C7B—H7BB	109.5
C6A—C5A—S1A	117.83 (16)	C2B—C7B—H7BC	109.5
O4A—C6A—O3A	125.4 (2)	H7BA—C7B—H7BC	109.5
O4A—C6A—C5A	119.7 (2)	H7BB—C7B—H7BC	109.5
O3A—C6A—C5A	114.8 (2)	C6B—C8B—H8BA	109.5
C2B—N1B—C6B	122.5 (2)	C6B—C8B—H8BB	109.5
C2B—N1B—H1B	121 (2)	H8BA—C8B—H8BB	109.5
C6B—N1B—H1B	116 (2)	C6B—C8B—H8BC	109.5
C2B—N3B—C4B	118.7 (2)	H8BA—C8B—H8BC	109.5
C4B—N4B—H4B1	118 (2)	H8BB—C8B—H8BC	109.5
C4B—N4B—H4B2	127 (2)		
			
O1A—C1A—C2A—C3A	−179.2 (2)	C4B—N3B—C2B—N1B	−1.3 (3)
O2A—C1A—C2A—C3A	2.2 (3)	C4B—N3B—C2B—C7B	178.83 (19)
O1A—C1A—C2A—S1A	3.9 (3)	C6B—N1B—C2B—N3B	−1.3 (3)
O2A—C1A—C2A—S1A	−174.75 (16)	C6B—N1B—C2B—C7B	178.6 (2)
C5A—S1A—C2A—C3A	0.03 (18)	C2B—N3B—C4B—N4B	−177.9 (2)
C5A—S1A—C2A—C1A	177.43 (18)	C2B—N3B—C4B—C5B	2.5 (3)
C1A—C2A—C3A—C4A	−177.4 (2)	N4B—C4B—C5B—C6B	179.2 (2)
S1A—C2A—C3A—C4A	−0.4 (3)	N3B—C4B—C5B—C6B	−1.2 (3)
C2A—C3A—C4A—C5A	0.6 (3)	N4B—C4B—C5B—Cl1B	−2.4 (3)
C3A—C4A—C5A—C6A	176.1 (2)	N3B—C4B—C5B—Cl1B	177.20 (17)
C3A—C4A—C5A—S1A	−0.6 (3)	C2B—N1B—C6B—C5B	2.4 (3)
C2A—S1A—C5A—C4A	0.31 (18)	C2B—N1B—C6B—C8B	−177.5 (2)
C2A—S1A—C5A—C6A	−176.79 (17)	C4B—C5B—C6B—N1B	−1.2 (3)
C4A—C5A—C6A—O4A	−162.8 (2)	Cl1B—C5B—C6B—N1B	−179.59 (16)
S1A—C5A—C6A—O4A	13.7 (3)	C4B—C5B—C6B—C8B	178.8 (2)
C4A—C5A—C6A—O3A	17.1 (3)	Cl1B—C5B—C6B—C8B	0.4 (3)
S1A—C5A—C6A—O3A	−166.44 (16)		

### Hydrogen-bond geometry (Å, º)

Cg is the centroid of the S1*A*/C2*A*–C5*A* ring.

**Table:**

*D*—H···*A*	*D*—H	H···*A*	*D*···*A*	*D*—H···*A*
O3*A*—H3*A*···O2*A*^i^	1.04 (4)	1.44 (4)	2.475 (2)	176 (4)
N1*B*—H1*B*···O1*A*	0.85 (3)	1.87 (3)	2.719 (3)	178 (3)
N4*B*—H4*B*1···N3*B*^ii^	0.86 (3)	2.40 (3)	3.218 (3)	158 (3)
N4*B*—H4*B*2···O4*A*^iii^	0.94 (3)	1.86 (3)	2.784 (3)	170 (3)
C7*B*—H7*BB*···S1*A*^iv^	0.98	2.86	3.807 (2)	164
C8*B*—H8*BB*···O3*A*^v^	0.98	2.53	3.281 (3)	134
C8*B*—H8*BC*···O2*A*^vi^	0.98	2.47	3.301 (3)	143
C7*B*—H7*BB*···*Cg*^iv^	0.98	2.69	3.556 (3)	148

Symmetry codes: (i) *x*+1/2, −*y*+3/2, *z*−1/2; (ii) −*x*+1, −*y*, −*z*+1; (iii) *x*−1/2, −*y*+1/2, *z*+1/2; (iv) −*x*+2, −*y*+1, −*z*+1; (v) *x*−1/2, −*y*+3/2, *z*+1/2; (vi) −*x*+3/2, *y*−1/2, −*z*+3/2.
